# Supplementary material for: Arabidopsis Class II Formins AtFH13 and AtFH14 Can Form Heterodimers but Exhibit Distinct Patterns of Cellular Localization
Source: Int J Mol Sci. 2020 Jan 5;21(1):348. doi: 10.3390/ijms21010348 (PMC6982070; doi:10.3390/ijms21010348)
Supplement: Supplementary file 1 [file ijms-21-00348-s001.pdf]

Supplementary data for Kollárová et al: Arabidopsis Class II formins AtFH13 and AtFH14 can form heterodimers but exhibit distinct patterns of cellular localization

The supplementary materials consist of the following:

Figure S1: Quantification of fluorescent protein-tagged AtFH13 and AtFH14 derivatives co-localization with other markers examined in this study (in this file),

Table S1: Summarization of cloning methods used for each entry clone (in this file).

Table S2: The combinations of entry clones and destination vectors used for generation of expression vectors used in this study (in this file).

Table S3: Summarization of primers, templates, destination vectors and restriction enzymes used to generate expression vectors for the yeast two hybrid assay (in this file).

Video S1: Co-expression of AtFH13-YFP with the microtubule marker KMD-RFP. Green: AtFH13-YFP. Magenta: KMD-RFP. Scale bar is 10 µm (in a separate file S1.avi; saved using 2x JPEG compression, frame rate 10 fps).

Video S2: Co-expression of AtFH14-YFP with the microtubule marker KMD-RFP. Green: AtFH14-YFP. Magenta: KMD-RFP. Scale bar is 10 µm (in a separate file S2.avi; saved using 2x JPEG compression, frame rate 10 fps).

Video S3: Co-expression of AtFH13-YFP with the endoplasmic reticulum marker ER-rk. Green: AtFH13-YFP. Magenta: ER-rk. Scale bar is 10 µm (in a separate file S3.avi; saved using 1x JPEG compression, frame rate 10 fps).

Video S4: Co-expression of AtFH14-YFP with the endoplasmic reticulum marker ER-rk. Green: AtFH14-YFP. Magenta: ER-rk.. Scale bar is 10 µm (in a separate file S4.avi; saved using 1x JPEG compression, frame rate 10 fps).

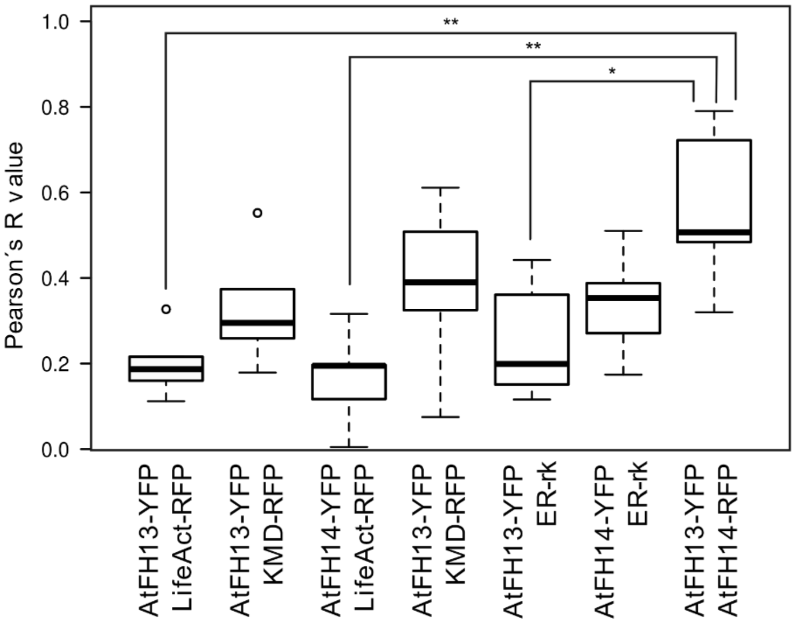

Figure S1: Quantification of fluorescent protein-tagged AtFH13 and AtFH14 derivatives co-localization with other markers examined in this study. Distribution of Pearson´s correlation coefficients for the indicated fluorescent protein pairs is shown. Asterisks indicate statistically significant difference (ANOVA): ** $p < 0.001$; * $p < 0.05$.

Table S1: Summarization of cloning methods used for each entry clone with specified primers, template, type of donor vector and restriction enzymes (in case of restriction cloning).In the primer sequence, the restriction enzyme sites are underlined with a continuous line and the adaptors for GW cloning are underlined with a dotted line.

Entry clone	Cloning method	Primer name	Primer sequence (5´-3´)	Template	Donor vector	Enzymes
**pEN_gAtFH13**	Restriction cloning	FH13PTEN_TAIR	ACTGGTACCAAATGGCATTGTTTCGCAAATTG	gDNA	pENTR™1A	KpnI
		FH13full_rev	GTCGGCCGAGGAGCGGTTCTTTCCTTTAG			EagI
**pEN_PTEN-FH13**	Restriction cloning	TAIR	ACTGGTACCAAATGGCATTGTTTCGCAAATTG	cDNA pACT library	pENTR™1A	KpnI
		PTENrev	CTTGCGGCCGATTTGCAATGGCTAACTGC			EagI
**pEN_PTENΔ-FH13**	Restriction cloning	TAIR	ACTGGTACCAAATGGCATTGTTTCGCAAATTG	cDNA pACT library	pENTR™1A	KpnI
		PTENrev	CTTGCGGCCGATTTGCAATGGCTAACTGC			EagI
**pEN_cAtFH14**	Restriction cloning	for_KpnI_CAMBIA14	TTGGTACCTTCCAATGTCTTTGTTAAG	cDNA AtFH14	pENTR™1A	KpnI
		rev_NotI_CAMBIA14	TTGCGGCCGCCATGTTCTATGTCTATGGATC			NotI
**pEN_FH2-FH14**	Restriction cloning	for_FH2/FH14_KpnI	TTGGTACCCCATGGGACTTGGAAGAG	cDNA AtFH14	pENTR™1A	KpnI
		rev_NotI_CAMBIA14	TTGCGGCCGCCATGTTCTATGTCTATGGATC			NotI
**pEN_PTEN-FH14**	Restriction cloning	FH14headKpn	TTGGTACCCTAACAAGGTTCTTCCAATG	gDNA	pENTR™1A	KpnI
		FH14headXho	GGACTCGAGGGAAGATTCAAAGGGTTG			XhoI
**pEN_FH2-FH13**	Gateway cloning	FH2dom_FH13attB1	GGGGACAAGTTTGTACAAAAAAGCAGGCTTAATGTTACGTGTTAATTTAAAGAATAGTCCAG	gDNA	pDONR221	none
		attB2AtFH13withoutSTOP	GGGGACCACTTTGTACAAGAAAGCTGGGTTAGGAGCGGTTCTTTCCTTTAG			

Table S2: The combinations of entry clones and destination vectors used for generation of expression vectors used in this study.

Expression vector	Entry clone	Destination vector
**AtFH13-YFP**	pEN_gAtFH13	pUBC-YFP-DEST
**FH2dom_FH13-GFP**	pEN_FH2-FH13	pUBC-GFP-DEST
**PTEN_FH13-YFP**	pEN_PTEN-FH13	pUBC-YFP-DEST
**PTENΔ_FH13-YFP**	pEN_PTENΔ-FH13	pUBC-YFP-DEST
**AtFH14-YFP**	pEN_cAtFH14	pUBC-YFP-DEST
**AtFH14-RFP**	pEN_cAtFH14	pUBC-RFP-DEST
**FH2dom_FH14-YFP**	pEN_FH2-FH14	pUBC-YFP-DEST
**PTEN_FH14-YFP**	pEN_PTEN-FH14	pUBC-YFP-DEST

Table S3: Summarization of primers, templates, destination vectors and restriction enzymes used to generatie expression vectors for the yeast two hybrid assay. In the primer sequences, restriction sites are underlined with a straight line.

Expression vector	Primer name	Primer sequence (5´-3´)	Template	Destination vector	Restriction enzymes
**AD:AtFH13**	AtFH13(FH2 domain) pGAD/pGBKT7 for	TTGAATTCGTTAATTTAAAGAATAGTCCAGCCG	pEN_FH2-FH13	pGADT7	EcoRI
	AtFH13(FH2 domain) pGAD/pGBKT7 rev	TTGGATCCGTTCTTTCCTTTAGTCGGTCAC			BamHI
**DBD:AtFH13**	AtFH13(FH2 domain) pGAD/pGBKT7 for	TTGAATTCGTTAATTTAAAGAATAGTCCAGCCG	pEN_FH2-FH13	pGBKT7	EcoRI
	AtFH13(FH2 domain) pGAD/pGBKT7 rev	TTGGATCCGTTCTTTCCTTTAGTCGGTCAC			BamHI
**AD::AtFH14**	AtFH14(FH2 domain) pGADT7 for	TTGAATTCAGGTTGGGTGCTCCCCCT	pEN_cAtFH14	pGADT7	EcoRI,
	AtFH14(FH2 domain) pGADT7 rev	TTCCCGGGTCTGCTGGATAAGATCGTTGTCGTT			XmaI
